# Image Classification of Alzheimer’s Disease Based on External-Attention Mechanism and Fully Convolutional Network

**DOI:** 10.3390/brainsci12030319

**Published:** 2022-02-26

**Authors:** Mingfeng Jiang, Bin Yan, Yang Li, Jucheng Zhang, Tieqiang Li, Wei Ke

**Affiliations:** 1 School of Information Science and Technology, Zhejiang Sci-Tech University, Hangzhou 310018, China; m.jiang@zstu.edu.cn (M.J.); yan0106air@163.com (B.Y.); 2 Department of Clinical Engineering, The Second Affiliated Hospital, School of Medicine, Zhejiang University, Hangzhou 310019, China; jucheng@zju.edu.cn; 3 Department of Clinical Science, Intervention and Technology, Karolinska Institutet, 171 77 Stockholm, Sweden; tie-qiang.li@ki.se; 4 School of Applied Sciences, Macao Polytechnic Institute, Macao SAR 999078, China; wke@ipm.edu.mo

**Keywords:** Alzheimer’s disease, fully convolutional network, external-attention mechanism, double normalization, image classification

## Abstract

Automatic and accurate classification of Alzheimer’s disease is a challenging and promising task. Fully Convolutional Network (FCN) can classify images at the pixel level. Adding an attention mechanism to the Fully Convolutional Network can effectively improve the classification performance of the model. However, the self-attention mechanism ignores the potential correlation between different samples. Aiming at this problem, we propose a new method for image classification of Alzheimer’s disease based on the external-attention mechanism. The external-attention module is added after the fourth convolutional block of the fully convolutional network model. At the same time, the double normalization method of Softmax and L1 norm is introduced to obtain a better classification performance and richer feature information of the disease probability map. The activation function Softmax can increase the degree of fitting of the neural network to the training set, which transforms linearity into nonlinearity, thereby increasing the flexibility of the neural network. The L1 norm can avoid the attention map being affected by especially large (especially small) eigenvalues. The experiments in this paper use 550 three-dimensional MRI images and use five-fold cross-validation. The experimental results show that the proposed image classification method for Alzheimer’s disease, combining the external-attention mechanism with double normalization, can effectively improve the classification performance of the model. With this method, the accuracy of the MLP-A model is 92.36%, the accuracy of the MLP-B model is 98.55%, and the accuracy of the fusion model MLP-C is 98.73%. The classification performance of the model is higher than similar models without adding any attention mechanism, and it is better than other comparison methods.

## 1. Introduction

Alzheimer’s disease (AD) is a progressively developing degenerative disease of the brain and nervous system. With the global escalation of the aging process, the incidence of Alzheimer’s disease is increasing every year. Elderly people with Alzheimer’s disease will experience a series of brain damages such as gradual memory loss, inconvenience of movement, decline in language expression and cognitive difficulties as the disease continues to worsen [1]. A large number of clinical studies have shown that drug intervention and care for early AD patients can delay the development of the disease and stabilize the patient’s condition. Therefore, the early and accurate judgment of patients with suspected AD has important practical significance.

At present, researchers use machine learning and deep learning to replace traditional methods for the auxiliary diagnosis of Alzheimer’s disease [2,3,4]. The methods of traditional machine learning for the classification of Alzheimer’s disease generally extract features from collected medical image data manually or semi-manually, and then send them to traditional classifiers for classification [5,6]. The algorithms of classification based on traditional methods mainly include two stages of feature extraction and classification, and sometimes also include feature selection and feature fusion [7,8,9]. In the process of image feature extraction, there are different methods such as Histogram of Oriented Gradient (HOG), Local Binary Pattern (LBP), and Principal Component Analysis (PCA) [10,11]. HOG constitutes a feature by calculating and counting the gradient direction histogram of the local area of the image. LBP is an operator used to describe the local texture features of the image; it has the advantages of rotation invariance and grayscale invariance. PCA is an effective algorithm for eliminating redundancy and simplifying datasets; it can remove redundant image features [12,13].

The deep learning uses the characteristics of its network to extract image features, discover hidden laws from it, and then achieve classification and recognition. Therefore, deep learning has achieved breakthrough results in target detection, face recognition, image classification and other fields [14,15,16,17]. In recent years, deep learning methods for Alzheimer’s disease classification have been continuously emerging, such as: the bottom-up unsupervised learning method of Stacked Auto Encoder (SAE), Deep Boltzmann Machine (DBM), and a top-down supervised learning method of deep convolutional neural network [18,19,20]. Suk et al. [21] used DBM to extract multi-modal features from PET and MRI data in the ADNI database, and used a 3D patch to pair potential hierarchical feature representations to classify AD and NC images; they got good results. Shi et al. [22] used a deep polynomial network (DPN) to classify AD and NC images of MRI and PET data respectively, and further proposed a multi-modal stacked deep polynomial network (MM-SDPN) to perform binary classification tasks, finally the accuracy of their experiment reached 96.93%. Recently, Tomassini et al. [23] proposed an end-to-end 3D convolutional long short-term memory network framework (LSTM) for early diagnosis of AD from full-resolution sMRI images.

In the process of Alzheimer’s disease research, the selection of different classifiers, model structures and appropriate attention mechanisms all play a crucial role in image classification, image recognition, and image segmentation [24]. For example, decision tree is a very common classification method [25]. It is a tree structure, each internal node represents a judgment on an attribute, each branch represents the output of a judgment result, and finally each leaf node represents a classification result. At the same time, in order to preserve the inherent characteristics of the original image and improve the good characteristics of the image in disease detection and classification, researchers usually use the latest visual sensing equipment, which can clearly observe tens of thousands of pixels in the image [26]. The vision sensor is the direct source of the machine vision system, it mainly consists of auxiliary equipment such as a graphics sensor and a light projector, which can obtain the original images that the machine vision system needs to process [27]. In addition, some researchers have studied the activation functions and pooling functions of the convolutional neural network, to compare the impact on the classification performance of Alzheimer’s disease [28,29]. Even image preprocessing is also an effective way to improve the classification performance of subsequent experiments, including template registration of images and various image filtering [30].

We conduct image classification of Alzheimer’s disease in order to better distinguish the difference between patients and normal people, and we are eager to apply it in clinical experiments in the future, but there are various uncertain problems in the research. Therefore, most researchers use neutrosophic statistics to expand and solve the uncertainty of various problems. Neutrosophic statistics refers to the statistical analysis of data samples with uncertainty, which is an extension of classical statistics and is suitable for situations where the data come from complex processes or uncertain environments [31].

In the field of computer vision, the attention mechanisms can effectively extract the feature of images. The attention mechanisms have various implementations, roughly divided into soft attention and hard attention [32,33,34]. The attention mechanism selects the focal position of the image, yielding more discriminative feature representations and bringing continuous performance improvements to the model. The soft attention mechanism means that when selecting information, it calculates the weighted average of the N input information instead of selecting only one information from the N information, and then inputs it into the neural network [35]. While the hard attention mechanism refers to selecting the information in a certain position of the input sequence, such as randomly selecting a piece of information or selecting the information with the highest probability. The visual attention mechanism can be used to pay attention to key areas in the image to obtain high-level information of image features.

The self-attention mechanism (SA) was proposed by Zhang et al. [36], and they used the weight matrix of three branches to capture the internal feature correlation of a single sample, thereby reducing the dependence on external information. But the self-attention mechanism has quadratic complexity and ignores the potential correlation between different samples. So, Guo et al. [37] proposed an external attention mechanism in 2021 to solve this problem, and they adopted two external matrices, M_k_ and M_v,_ to model the potential correlation between samples. Meanwhile, the external attention mechanism has linear complexity and implicitly considers the correlation among all data samples. Recently, Jiao et al. [38] proposed a feature fusion model for AD classification, which can comprehensively utilize multiple types of data to improve the classification performance.

Based on the fully convolutional network, this paper proposes a new method for image classification of Alzheimer’s disease that combines the external-attention mechanism with double normalization [39]. First, we obtain the feature information of the disease probability map through the FCN model, and then select the region of interest (ROI) according to the MCC heatmap of the FCN model, finally combine with age, gender, MMSE as the input of the MLP model to classify AD and NC images. The contributions of this paper are: (1) We propose a method for image classification of Alzheimer’s disease based on external-attention mechanism and fully convolutional network; (2) and add a self-attention module to the FCN model as a comparative experiment to highlight the effectiveness and efficiency of the external-attention mechanism; (3) In the normalization process of the attention map, the double normalization method of Softmax and L1 norm is used to replace the original Softmax, which can improve the classification performance in a small range.

## 2. Materials and Methods

### 2.1. Datasets

In order to prove the effectiveness of the self-attention mechanism and the external-attention mechanism, this paper uses T1-weighted Alzheimer’s disease MRI images in the ADNI dataset for experiments. The specific details of the dataset are shown in Table 1. The dataset of the experiments selects individual scan images over 55 years old, which includes a total of 550 1.5 T three-dimensional MRI images, of which 307 images belong to Alzheimer’s disease (AD) patients and 243 images belong to normal cognitive persons.

### 2.2. Image Preprocessing

We use the FLIRT tool in the FSL software package to align the brain magnetic resonance image with the MNI152 public template. We use the matrix that the image is invariant to affine transformation to determine the parameters of the transformation function, and then transform the original image into a standard form of image according to the transformation function determined by this parameter, finally the image size is 182 × 218 × 182. FLIRT uses coordinate rotation, translation, scaling, and shearing to match two images together, and the cost function O(w) is expressed in the form of quadratic summation, the intensity difference between input image and the public template is used as the optimization objective.

(1) $$O\left(w\right)=\sum_{i=1}^{N}{\left(g\left(x_{i}^{\prime }\left(x_{i},w\right)\right)-f\left(x_{i}\right)\right)}^{2},$$

$f\left(x_{i}\right)$ represents the intensity of the public template, $g\left(x_{i}\right)$ represents the intensity of the input image, $x_{i}^{\prime }$ represents the value of $x_{i}$ after affine transformation.

**Figure 1:**
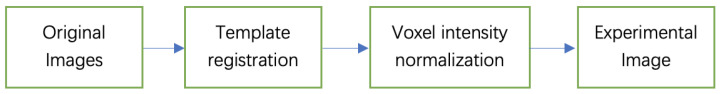
The flowchart of image preprocessing.

After the image is registered, we normalize the voxel values of all images. Then we control these voxel values and other outliers within a certain range to avoid the interference of background information. The flowchart of image preprocessing is shown in Figure 1.

### 2.3. Experimental Settings and Evaluation Criteria

The experiments in this paper use the Pytorch deep learning framework and GeForce RTX 3090 GPU processor. The FCN model uses Adam optimizer and cross-entropy loss function. In addition, the experimental parameters are set: batch size is 10, learning rate is 0.0001, and the number of training iterations is 3000. The validation set is verified every 20 iterations, then the optimal model and weights are saved. Finally, the optimal model is tested with the test set to obtain the classification performance of the FCN model and the feature information of the disease probability map.

We use accuracy and Matthews correlation coefficient (MCC) to evaluate the classification performance of the FCN model. In addition, we also record the age, gender and MMSE of AD patients and normal cognitive persons in this dataset, as the input of the MLP model’s classification experiments. As for the MLP model, we use accuracy (marked as Accu), sensitivity (marked as Sens), specificity (marked as Spec), F1 score and MCC to evaluate its classification performance. F1 score is an indicator used to measure the two-class model in statistics. It takes into account the accuracy and recall of the classification model at the same time, and can be regarded as the harmonic average of the model’s accuracy and recall. MCC comprehensively considers true positives, true negatives, false positives, and false negatives, and is a relatively balanced indicator in deep learning. In order to ensure the accuracy and reliability of the experiments, we use five-fold cross-validation for the experiments, repeat the experiments five times for the FCN model, and three times for the MLP model. The final classification performance of the models is represented by the mean and standard deviation.

(2) $$Accu=\frac{TP+TN}{TP+TN+FP+FN}$$

(3) $$Sens=\frac{TP}{TP+FN}$$

(4) $$Spec=\frac{TN}{TP+FP}$$

(5) $$F1=\frac{2TP}{2TP+FN+FP},$$

(6) $$MCC=\frac{TP\times TN-FP\times FN}{{\left[\left(TP+FP\right)\left(TP+FN\right)\left(TN+FP\right)\left(TN+FN\right)\right]}^{0.5}}.$$

Among them, true positives (*TP*) represent the correct predictions of positive samples, true negatives (*TN*) represent the correct predictions of negative samples, false negatives (*FN*) represent the false predictions of positive samples, and false positives (*FP*) represent the false predictions of negative samples.

### 2.4. Methods

#### 2.4.1. Self-Attention Mechanism

**Figure 2:**
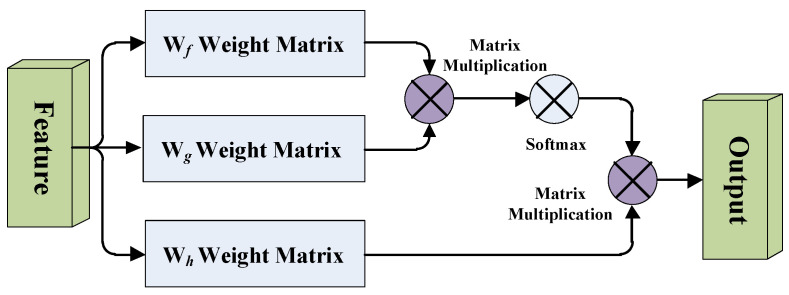
Diagram of the self-attention mechanism.

A simplified diagram of the self-attention mechanism is shown in Figure 2. W*_f_*, W*_g_* and W*_h_* are the weight matrices of the 1 × 1 × 1 convolutional layer, in order to learn the local dependencies of the image, and it also learns the long-distance global dependencies.

We take the feature map after the last convolutional block of the FCN model, passing through three branches of the 1 × 1 × 1 convolutional layers *f*(*x*), *g*(*x*), *h*(*x*) and the number of channels of the three branches is C, where H, W and D represent the length, width, and depth of the feature map respectively. After that, we transpose the output of matrix *f*(*x*) and multiply it with the output of matrix *g*(*x*), then multiply it by Softmax for normalization to obtain the attention feature map. Finally, we multiply the attention feature map with the output of matrix *h*(*x*), then pass it through the 1 × 1 × 1 convolutional layer to integrate the output into a self-attention feature map.

(7) $$f\left(x\right)=W_{f}x,$$

(8) $$g\left(x\right)=W_{g}x,$$

(9) $$h\left(x\right)=W_{h}x,$$

$x\in {\mathbb{R}}^{C\times D\times H\times W}$ is the original feature map before the input of three branches.

(10) $$s_{ij}=f{\left(x_{i}\right)}^{T}g\left(x_{j}\right),$$

(11) $$\beta_{j,i}=\frac{\exp\left(s_{ij}\right)}{\sum_{i=1}^{N}\exp\left(s_{ij}\right)},$$

$f\left(x_{i}\right)$ means the values of all channels at the *i*-th pixel position, $g\left(x_{j}\right)$ means the values of all channels at the *j*-th pixel position. $\beta_{j,i}$ means the degree of attention of the model to the *i*-th region when synthesizing the *j*-th region.

The output of the self-attention module is defined as:

(12) $$O_{j}=v\left(\sum_{i=1}^{N}\beta_{j,i}h\left(x_{i}\right)\right),v\left(x_{i}\right)=W_{v}x_{i},$$

We multiply the output $O_{j}$ of the self-attention module with a weight coefficient γ and then add it to the input feature map $x_{i}$ to get the final output $y_{i}$ of the self-attention module:

(13) $$y_{i}=\gamma O_{j}+x_{i}.$$

Among them, γ is a learnable parameter, and its function is to enable the network to learn the proportion of global dependence in the feature map by itself.

**Figure 3:**
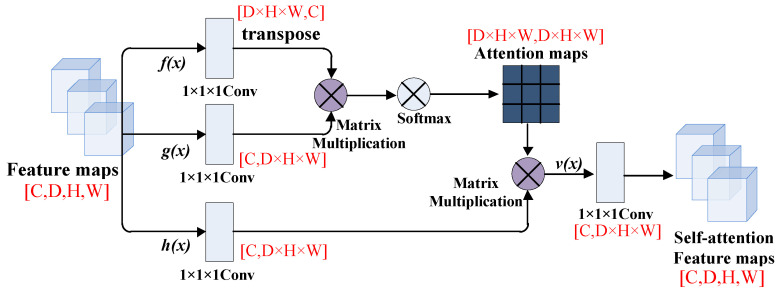
The feature maps in the self-attention module.

The tensor dimension’s changes of the feature map in the self-attention module are shown in Figure 3. In the process of feature map convolution, in order to make each eigenvalue in the image interact with each other, we change the number of channels and matrix dimensions of the convolution. D × H × W means the total pixels of the feature map.

#### 2.4.2. External-Attention Mechanism

**Figure 4:**
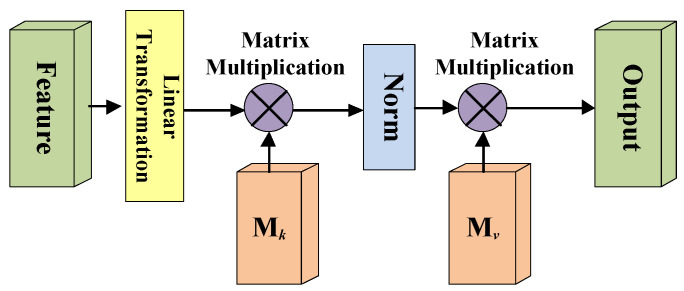
Diagram of the external-attention mechanism.

A simplified diagram of the external attention mechanism is shown in Figure 4. Here, the linear transformation refers to the 1 × 1 × 1 convolutional layer processing, and the Norm means double normalization; $M_{k}$ and $M_{v}$ are two external matrices.

Due to the self-attention mechanism only considering the value of a single sample to make the attention map, so the external attention mechanism uses two external matrices $M_{k}$ and $M_{v}$ which are different from the self-attention mechanism ($M_{k}$ and $M_{v}$ are linear layer without bias), to model the similarity between the *i*-th pixel and the *j*-th pixel. Of course, the matric *M* is learnable, and it can also model the potential connections between different samples in the whole dataset as the training process progresses.

(14) $$A=Norm\left(FM_{k}^{T}\right),$$

(15) $$F_{out}=AM_{v}.$$

$F\in {\mathbb{R}}^{C\times D\times H\times W}$ is the output feature map of the last convolutional block, Norm means to normalize $FM_{k}^{T}$ and $F_{out}$ is the output feature map of the FCN model after adding the external-attention module.

In the self-attention module, Softmax is used to normalize the attention map to make $\Sigma_{j}\alpha_{i,j}=1$. However, the attention map is calculated by matrix multiplication, which is very sensitive to the size of the input feature. When a certain eigenvalue is very large or very small, its dot product to other eigenvalues will also become very large or very small. Therefore, the external attention module uses the double normalization of Softmax and L1 norm to solve this problem, that is, first applies Softmax to the columns of the attention map, and then applies L1 norm to the rows.

(16) $${\tilde{a}}_{i,j}=FM_{k}^{T},$$

(17) $${\hat{\alpha }}_{i,j}=\frac{\exp\left({\tilde{a}}_{i,j}\right)}{\Sigma_{k}\exp\left({\tilde{a}}_{k,j}\right)},$$

(18) $$\alpha_{i,j}=\frac{{\hat{\alpha }}_{i,j}}{\Sigma_{k}{\hat{\alpha }}_{i,k}}.$$

${\tilde{a}}_{i,j}$ represents the input feature map F multiplying with the transposed external matrix $M_{k}$. ${\hat{\alpha }}_{i,j}$ represents normalizing the columns of the attention map ${\tilde{a}}_{i,j}$ by Softmax, and $\alpha_{i,j}$ represents normalizing the rows of the attention map ${\hat{\alpha }}_{i,j}$ by L1 norm. Among them, the *k* of ${\tilde{a}}_{k,j}$ and ${\hat{\alpha }}_{i,k}$ represents the number of channels in the linear layer.

**Figure 5:**
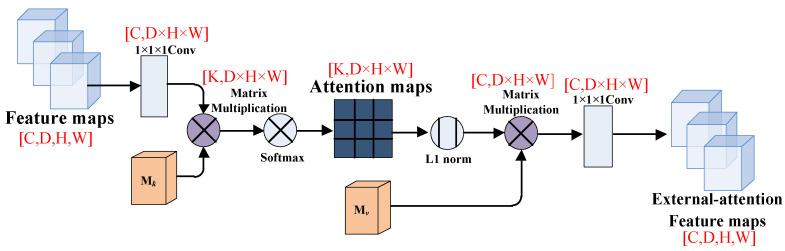
The feature maps in the external-attention module.

The tensor dimension’s changes of the feature map in the external attention module are shown in Figure 5. First, we use 1 × 1 × 1 convolutional layer to change the tensor dimension, and then multiply it with the transposed external matrix $M_{k}$. The *k* means the number of channels of the external matrix and the D × H × W means the total pixels of the feature map. After that, we apply the double normalization to normalize the attention map. Then, we multiply the attention map with the external matrix $M_{v}$ to put it into a new 1 × 1 × 1 convolutional layer. Finally, we can obtain the external-attention feature map with the same tensor dimension as the original feature map.

#### 2.4.3. Model’s Framework

**Figure 6:**
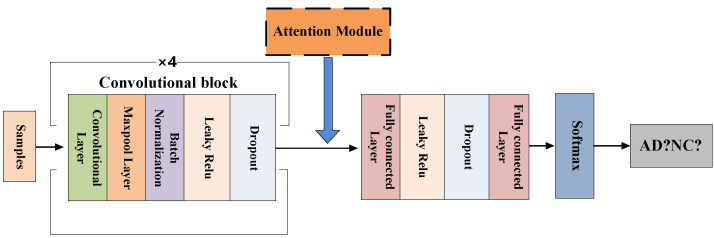
The FCN model’s framework.

**Figure 7:**
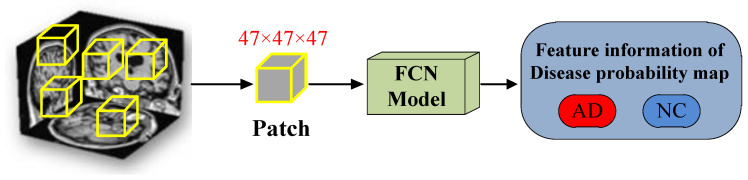
Randomly sampling 3D-MRI image’s patches for training the FCN Model.

The FCN model consists of four convolutional blocks and two fully connected layers. Among them, the convolutional block includes 3D convolutional layer, 3D maxpool layer, 3D batch normalization, Leaky ReLU and Dropout, as shown in the Figure 6. The last two fully connected layers play a role in improving the efficiency of the model in the classification task. The network is trained by randomly initializing weights. As shown in Figure 7, we adopt a method of randomly sampling patches of the 3D-MRI images to train the FCN model, that is, training patches with a random sampling size of 47 × 47 × 47 from the 3D-MRI images. And the size of each patch is the same as the receptive field of FCN model.

Because convolution operation reduces the output size of the input layer, each Patch will generate two scalar values after being trained by the FCN model. Then they are converted into the Alzheimer’s disease probability and normal cognitive probability of the corresponding pixel under the action of the activation function Softmax. The disease state of the brain’s local structure is displayed through each pixel’s risk probability value of Alzheimer’s disease. The corresponding feature information of the disease probability map will be used as auxiliary information of the MLP model for classification experiments.

**Figure 8:**
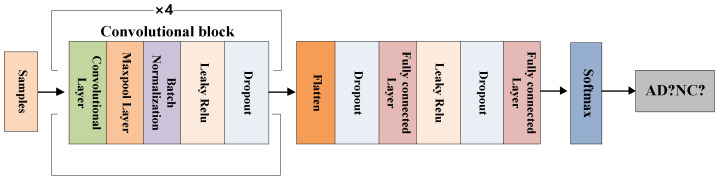
The CNN model’s framework.

In order to conduct comparative experiments with the FCN model, we also use the same network for the CNN model, as shown in Figure 8. The specific parameter settings of the hidden layer of the CNN model are shown in Table 2.

**Figure 9:**
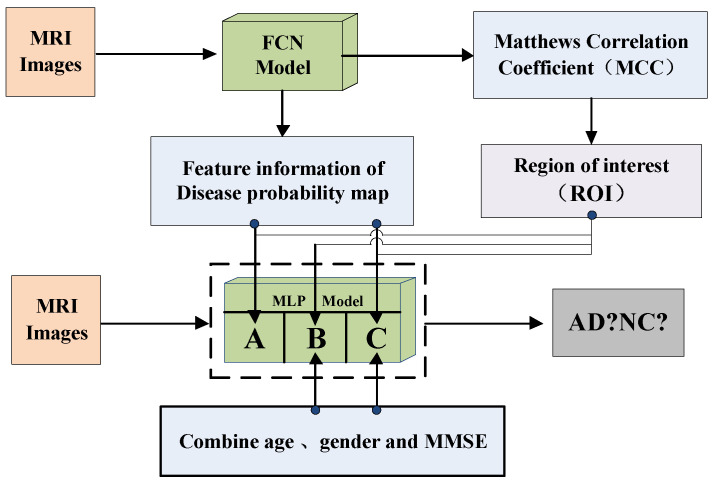
The MLP model’s framework.

After that, we build the MLP model’s framework, as shown in Figure 9. The MLP model consists of two fully connected layers; batch normalization, Leaky ReLU and Dropout. For the MLP model, we select the probability value of Alzheimer’s disease from the feature information of the disease probability map, and select the region of interest (ROI) based on the MCC value of the FCN model, then combine with the age, gender, MMSE of the MRI image as the input of the MLP model to reclassify MRI images.

Among them, the MCC value can show the overall classification performance of the FCN model, and the MCC heatmap can show that the FCN model has a higher classification accuracy for certain pixel positions of the 3D-MRI image. Therefore, we select these high-accuracy regions as regions of interest (ROI).

According to Figure 9, the MLP-A model indicates that only the feature information of the disease probability map of the FCN model is used to classify MRI images; The MLP-B model indicates that only the image information of age, gender and MMSE are used to classify MRI images; The MLP-C model indicates combining the feature information of the disease probability map of the FCN model with age, gender, MMSE to classify MRI images.

In addition, the specific FCN model’s parameter settings and the changes of output patch size in our experiments are shown in the Table 3.

## 3. Experiments and Results

**Figure 10:**
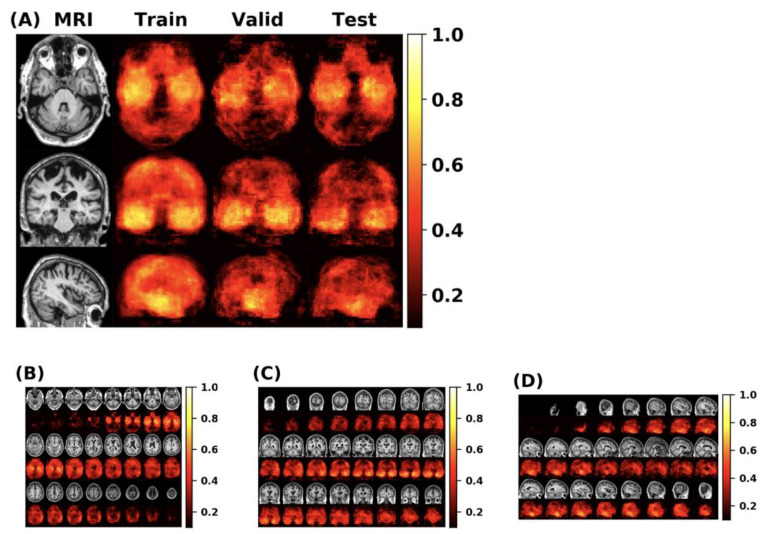
(**A**) The MCC value can show the overall classification performance of the FCN model. From the MCC heatmap, it can be observed that some locations have higher MCC values (that is, these locations have higher classification accuracy). The MLP model uses these specific locations as a region of interest (ROI). (**B**–**D**) represents the MCC value of the FCN model in the individual axial, coronal and sagittal directions.

First, this paper conducts experiments on the original FCN model and MLP model, then adds the self-attention module and the external-attention module respectively for multiple experiments. Finally, we use the mean and standard deviation to represent the classification performance of the model. The double normalization of Softmax and L1 norm are used to replace Softmax in the external-attention module. The MCC heatmap of the FCN model is shown in Figure 10.

**Figure 11:**
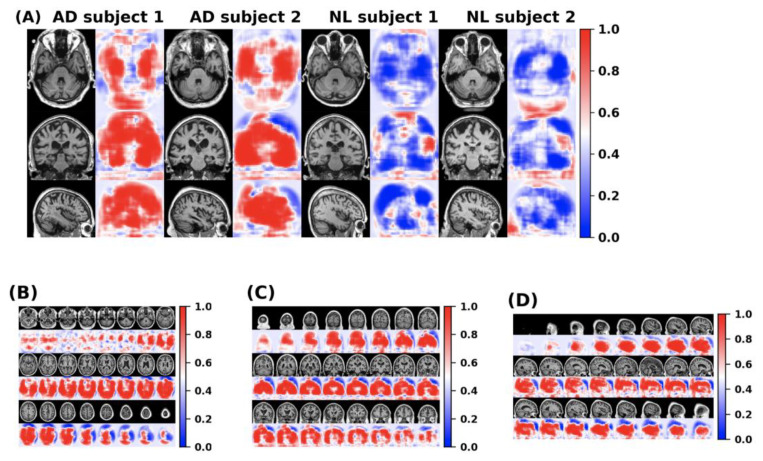
(**A**) The disease probability map generated by the FCN model highlights the brain regions at high risk of Alzheimer’s disease. The first two samples were clinically diagnosed as patients with Alzheimer’s disease, and the latter two samples were clinically confirmed as normal cognitive persons. (**B**–**D**) shows the axial, coronal and sagittal disease probability map of patients who are clinically diagnosed with Alzheimer’s disease. Red indicates that the risk of Alzheimer’s disease is >0.5, and blue indicates <0.5.

The feature information of the disease probability map generated by the FCN model is shown in the Figure 11. Red and blue indicate the probability of suffering from Alzheimer’s disease in different parts of the brain. The dividing line between the two is 0.5.

**Figure 12:**
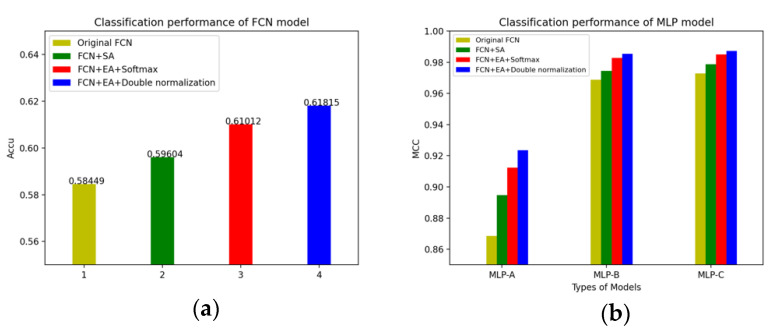
(**a**) The changes of the FCN models’ accuracy; (**b**) The changes of the MLP models’ accuracy.

We have summarized the changes in the accuracy of the FCN models and the MLP models in different situations. The changes of the FCN models’ accuracy and the MLP models’ accuracy after adding the self-attention mechanism and the external-attention mechanism are shown in Figure 12.

**Figure 13:**
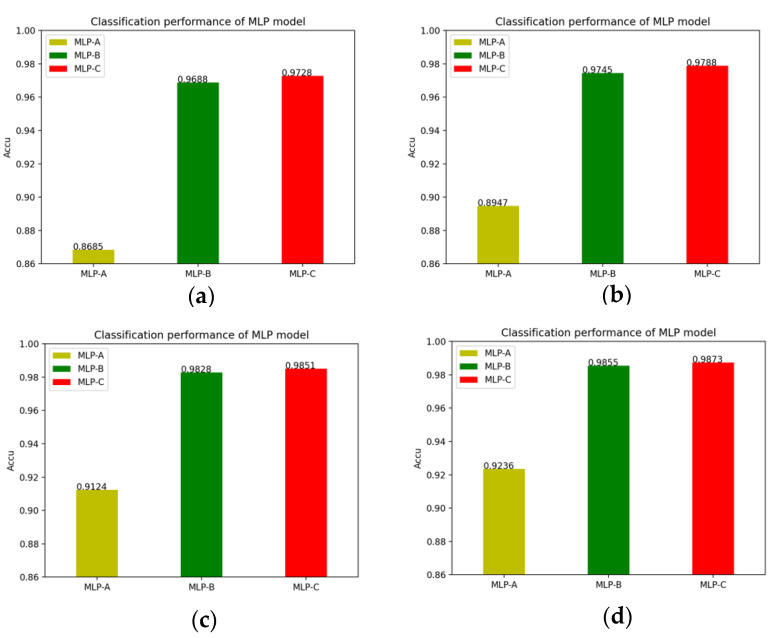
(**a**) No attention mechanism is added to models; (**b**) Models add self-attention mechanism; (**c**) Models add external-attention mechanism and Softmax; (**d**) Models add external-attention mechanism and double normalization.

In detail, the changes of the MLP models’ accuracy after adding the self-attention mechanism and the external-attention mechanism, as well as the double normalization are shown in Figure 13.

**Figure 14:**
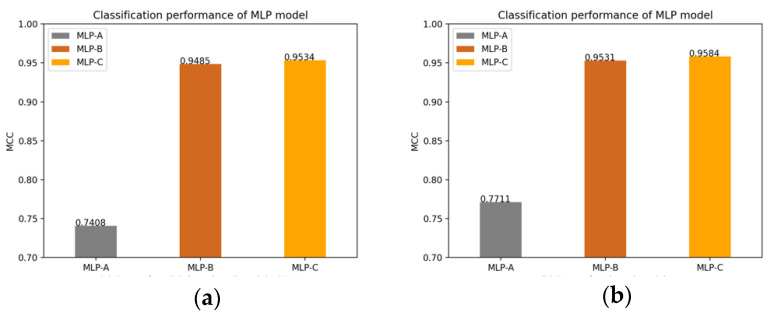

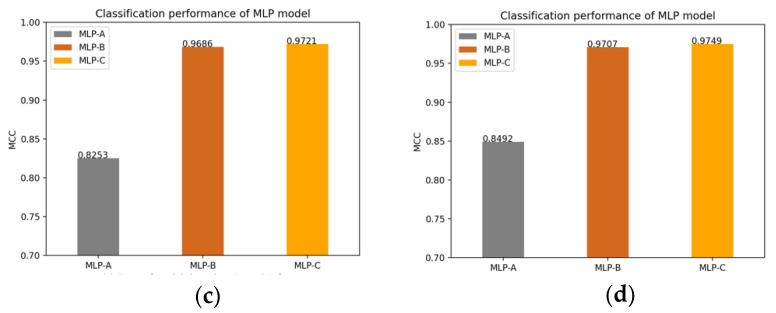
(**a**) Models without attention mechanism; (**b**) Models add self-attention mechanism; (**c**) Models add external-attention mechanism and Softmax; (**d**) Models add external-attention mechanism and double normalization.

Of course, we also plot the changes of the MLP models’ MCC value after adding the self-attention mechanism, the external attention mechanism and double normalization, as shown in Figure 14.

Next, we list the various experimental results of the FCN models and the MLP models, which are the final classification performance (mean and standard deviation), including accuracy, sensitivity, specificity, F1 score, MCC. The classification performance of the FCN model without any attention module is shown in Table 4.

As a comparative experiment, the experimental results of the CNN model and the MLP fusion model are shown in Table 5. Among them, the fusion model means that MLP model combines the feature information of the CNN model with age, gender, and MMSE to classify MRI images.

Comparing the experimental results of the MLP-C in Table 4 with the fusion model in Table 5. It can be found that selecting the region of interest (ROI) of the MLP model through the MCC heatmap of the FCN model, and combining with the feature information of the disease probability map can improve the classification performance better than the MLP fusion model.

We use accuracy and MCC to evaluate the classification performance of the FCN model, as shown in Table 6.

At here, the MCC is calculated by using each pixel in the 3D-MRI image as a sample. After each pixel in the 3D-MRI image is trained by the FCN model, a predicted probability value of Alzheimer’s disease will be generated. The prediction of each pixel is compared with the input label, and then the corresponding pixel is marked as TP, TN, FP, FN.

The classification performance of the MLP models after adding the self-attention module is shown in Table 7.

The results in Table 6 show that after the self-attention module is integrated into the FCN model, the accuracy increases of about 1.155%, and the MCC value increases of about 2.454%. Comparing the experimental results in Table 4 and Table 7, it can be found that after adding the self-attention module, for the MLP models, the accuracy increases by about 0.57% to 2.62%, and the MCC value increases by about 0.60% to 3.03%. This shows the effectiveness of the self-attention mechanism and it can improve the classification performance of the model.

On the other hand, the experimental results in Table 6 show that after adding the external-attention module and double normalization, compared with the original FCN model, the accuracy increases by about 3.366%, and the MCC value increases by about 5.195%. Furthermore, double normalization compares with Softmax, the accuracy increases by about 0.803%, and the MCC value increases by about 0.432%.

After adding the external-attention module, the classification performance of the MLP models by using double normalization or Softmax respectively are shown in Table 8 and Table 9.

Table 8 and Table 9 compare the classification performance difference between using double normalization with only using Softmax in the external-attention mechanism. Experimental results show that double normalization can increase the accuracy of the MLP models by about 0.22% to 1.12%, and the MCC value by about 0.21% to 2.39%. This shows that double normalization can improve the classification performance in a small range, highlighting the effectiveness of double normalization.

The classification index sensitivity of the model represents the proportion of all positive samples that are paired and measures the model’s ability to discriminate against positive samples. From the experimental results, after adding an external-attention mechanism to the FCN model and combining with double normalization, the sensitivity of MLP-A model classification is 92.6%, the sensitivity of MLP-B model classification is 99.02%, and the sensitivity of MLP-C model classification is 99.29%. This also shows that our proposed model has a high discriminative ability for images of Alzheimer’s disease patients.

## 4. Discussion

The above experimental results show that the external-attention mechanism can generate richer feature information of a disease probability map for the MLP models, thereby improving the classification performance of the model. In addition, the MLP models combine with age, gender, and MMSE, which are more conducive to the accurate judgment of image classification, in the case of selecting the region of interest (ROI) according to the FCN model.

The reason we choose to add the self-attention module to the FCN model as a comparative experiment is because the external-attention mechanism changes the weight matrix on the basis of the self-attention mechanism. The self-attention mechanism calculates the direct interaction between any two locations, allowing the network to focus on areas that are scattered in different locations. However, this self-attention mechanism only considers the correlations within a single sample, it ignores the potential connections between samples. Therefore, the external-attention mechanism uses a learnable external matrix to establish potential correlations between samples. In addition, the Softmax in the self-attention module normalizes the attention map, but the attention map is calculated by matrix multiplication, which is sensitive to the size of the input feature and susceptible to particularly large or small feature values. Therefore, the double normalization in the external-attention module first applies Softmax to the columns, and then applies L1 norm to the rows to solve this problem. The experimental results show that after adding external-attention mechanism to the fully convolutional network and combining with double normalization, the classification performance of the MLP models is better than other comparison methods. However, due to the many unknown factors of the deep learning model, the limitation of our study is that it is difficult to quantify and visually analyze the correspondence between models and results, which makes it difficult to apply in clinical practice. Therefore, we expect further research on the interpretability of the deep learning model in order to improve the confidence of the classification results.

Finally, we compare with the models of other references on Alzheimer’s disease, as shown in Table 10. Different classification techniques have their own advantages and disadvantages. For example, SVM uses the inner product kernel function to replace the nonlinear mapping to the high-dimensional space, and its goal is to divide the feature space into the optimal classification hyperplane [40]. However, its disadvantage is that it is difficult to implement large-scale training samples, and it is sensitive to the choice of parameter adjustment and function.

## 5. Conclusions and Future Work

In this paper, the self-attention mechanism models the correlations within the samples to obtain the corresponding attention feature maps, which plays a certain role in improving the classification performance of the model. Moreover, this paper proposes a new method for the image classification of Alzheimer’s disease based on the external-attention mechanism and double normalization, which embeds the external-attention module after the last convolutional block of the FCN model. The detailed experimental results show that the external-attention mechanism is effective and efficient in improving classification performance. We used the double normalization of Softmax and L1 norm to replace the original Softmax, so that the classification accuracy of the MLP model can increase by about 0.22% to 1.12%. The core of this paper is to integrate the external-attention mechanism into the FCN model to obtain richer and more detailed feature information of a disease probability map.

In the future, we will consider using other deep learning methods or new efficient attention mechanisms to continuously tap the potential of fully convolutional networks. In the image preprocessing, we will try to use different image denoising and smoothing methods to effectively remove noise from the original MRI image, and further improve the classification performance of the model. In addition, we will try different experimental approaches using 2D slices and 3D patches to compare the classification performance of the two [43], which has special implications for Alzheimer’s disease classification research.

## Figures and Tables

**Table 1:** Detailed information of the experimental dataset.

Dataset	ADNI	
Research object	AD	NC
Number of samples	307	243
Average age	76.3 (57–92)	79.4 (65–87)
Gender (Male/Female)	180/127	101/142
Average MMSE	22.8 (19–27)	28.6 (26–30)

**Table 2:** The parameter settings of CNN model.

Input Layer	Detailed Description	Output Size
Input		(1, 182, 218, 182)
3D convolutional layer1	channel 20, kernel 7, stride 2, padding 0	(20, 88, 106, 88)
3D maxpool layer1	kernel 3, stride 2, padding 0	(20, 43, 52, 43)
3D batch normalization1	eps = 1 × 10^−5^, momentum = 0.1, affine = True	
Leaky ReLU1; Dropout1	Negative slope = 0.01; *p* = 0.1	
3D convolutional layer2	channel 40, kernel 4, stride 1, padding 0	(40, 40, 49, 40)
3D maxpool layer2	kernel 2, stride 2, padding 0	(40, 20, 24, 20)
3D batch normalization2	eps = 1 × 10^−5^, momentum = 0.1, affine = True	
Leaky ReLU2; Dropout2	Negative slope = 0.01; *p* = 0.1	
3D convolutional layer3	channel 80, kernel 3, stride 1, padding 0	(80, 18, 22, 18)
3D maxpool layer3	kernel 2, stride 2, padding 0	(80, 9, 11, 9)
3D batch normalization3	eps = 1 × 10^−5^, momentum = 0.1, affine = True	
Leaky ReLU3; Dropout3	Negative slope = 0.01; *p* = 0.1	
3D convolutional layer4	channel 160, kernel 3, stride 1, padding 0	(160, 7, 9, 7)
3D maxpool layer4	kernel 2, stride 1, padding 0	(160, 6, 8, 6)
3D batch normalization4	eps = 1 × 10^−5^, momentum = 0.1, affine = True	
Leaky ReLU4; Dropout4	Negative slope = 0.01; *p* = 0.1	
Flatten		(46, 80)
Dropout5	*p* = 0.1	
Fully connected layer1	channel 30	(30)
Leaky ReLU5; Dropout6	Negative slope = 0.01; *p* = 0.1	
Fully connected layer2	channel 2	(2)

**Table 3:** The parameter settings of FCN model.

Input Layer	Detailed Description	Output Patch Size
Input		(1, 47, 47, 47)
3D convolutional layer1	channel 20, kernel 4, stride 1, padding 0	(20, 44, 44, 44)
3D maxpool layer1	kernel 2, stride 1, padding 0	(20, 43, 43, 43)
3D batch normalization1	eps = 1 × 10^−5^, momentum = 0.1, affine = True	
Leaky ReLU1; Dropout1	Negative slope = 0.01; *p* = 0.1	
3D convolutional layer2	channel 40, kernel 4, stride 1, padding 0	(40, 40, 40, 40)
3D maxpool layer2	kernel 2, stride 2, padding 0	(40, 20, 20, 20)
3D batch normalization2	eps = 1 × 10^−5^, momentum = 0.1, affine = True	
Leaky ReLU2; Dropout2	Negative slope = 0.01; *p* = 0.1	
3D convolutional layer3	channel 80, kernel 3, stride 1, padding 0	(80, 18, 18, 18)
3D maxpool layer3	kernel 2, stride 2, padding 0	(80, 9, 9, 9)
3D batch normalization3	eps = 1 × 10^−5^, momentum = 0.1, affine = True	
Leaky ReLU3; Dropout3	Negative slope = 0.01; *p* = 0.1	
3D convolutional layer4	channel 160, kernel 3, stride 1, padding 0	(160, 7, 7, 7)
3D maxpool layer4	kernel 2, stride 1, padding 0	(160, 6, 6, 6)
3D batch normalization4	eps = 1 × 10^−5^, momentum = 0.1, affine = True	
Leaky ReLU4; Dropout4	Negative slope = 0.01; *p* = 0.1	
Fully connected layer1	channel 30, kernel 6, stride 1, padding 0	(30, 1, 1, 1)
Leaky ReLU5; Dropout5	Negative slope = 0.01; *p* = 0.1	
Fully connected layer2	channel 2, kernel 1, stride 1, padding 0	(2, 1, 1, 1)

**Table 4:** The classification performance of the MLP models without any attention module.

FCN	Accu	Sens	Spec	F1	MCC
MLP-A	0.8685 ± 0.0140	0.8444 ± 0.0356	0.8943 ± 0.0481	0.8693 ± 0.0131	0.7408 ± 0.0288
MLP-B	0.9688 ± 0.0103	0.9526 ± 0.0221	0.9642 ± 0.0175	0.9691 ± 0.0104	0.9485 ± 0.0197
MLP-C	0.9728 ± 0.0143	0.9643 ± 0.0180	0.9674 ± 0.0139	0.9757 ± 0.0131	0.9534 ± 0.0177

**Table 5:** The experimental results of the CNN model and the MLP fusion model.

	Accu	Sens	Spec	F1	MCC
CNN	0.8636 ± 0.0237	0.8875 ± 0.0153	0.8696 ± 0.0364	0.8287 ± 0.0488	0.7549 ± 0.0368
MLP fusion model	0.9188 ± 0.0221	0.9439 ± 0.0340	0.8918 ± 0.0280	0.9232 ± 0.0215	0.8389 ± 0.0446

**Table 6:** The classification performance of the FCN model.

	Accu	MCC
FCN	0.58449 ± 0.0129	0.16132 ± 0.0264
FCN + SA	0.59604 ± 0.0052	0.18586 ± 0.0076
FCN + EA + Softmax	0.61012 ± 0.0105	0.20895 ± 0.0090
FCN + EA + Double normalization	0.61815 ± 0.0069	0.21327 ± 0.0138

**Table 7:** The classification performance of the MLP models after adding the self-attention module.

FCN + SA	Accu	Sens	Spec	F1	MCC
MLP-A	0.8947 ± 0.0103	0.8696 ± 0.0162	0.9107 ± 0.0134	0.8846 ± 0.0123	0.7711 ± 0.0189
MLP-B	0.9745 ± 0.0101	0.9649 ± 0.0248	0.9689 ± 0.0220	0.9751 ± 0.0100	0.9531 ± 0.0193
MLP-C	0.9788 ± 0.0113	0.9704 ± 0.0241	0.9712 ± 0.0152	0.9792 ± 0.0113	0.9584 ± 0.0219

**Table 8:** The classification performance of the MLP models after adding the external-attention module and double normalization.

FCN + EA + Double Normalization	Accu	Sens	Spec	F1	MCC
MLP-A	0.9236 ± 0.0193	0.9260 ± 0.0141	0.9356 ± 0.0163	0.9292 ± 0.0182	0.8492 ± 0.0180
MLP-B	0.9855 ± 0.0045	0.9902 ± 0.0080	0.9796 ± 0.0129	0.9869 ± 0.0040	0.9707 ± 0.0090
MLP-C	0.9873 ± 0.0069	0.9929 ± 0.0041	0.9828 ± 0.0185	0.9889 ± 0.0076	0.9749 ± 0.0130

**Table 9:** The classification performance of the MLP models after adding the external-attention module and Softmax.

FCN + EA + Softmax	Accu	Sens	Spec	F1	MCC
MLP-A	0.9124 ± 0.0112	0.9116 ± 0.0111	0.9234 ± 0.0156	0.9111 ± 0.0100	0.8253 ± 0.0140
MLP-B	0.9828 ± 0.0147	0.9867 ± 0.0089	0.9701 ± 0.0246	0.9836 ± 0.0129	0.9686 ± 0.0296
MLP-C	0.9851 ± 0.0099	0.9906 ± 0.0072	0.9741 ± 0.0204	0.9876 ± 0.0088	0.9721 ± 0.0198

**Table 10:** Compare with the classification models of other researchers.

Author	Type of Dataset	Methods	Number of Samples	Accuracy
Liu S et al. [20]	MRI	Stacked auto-encoder (SAE) + region-level engineered features	180 AD/204 NC	0.79
Shi J et al. [22]	MRI	Deep Polynomial Network (DPN)	51 AD/52 NC	0.9076
Tomassini S et al. [23]	MRI	Based on long short-term memory network (LSTM)	213 AD/214 NC	0.86
Ullah H et al. [41]	MRI	Deep Convolutional Network (3D-CNN)	416 (AD + NC)	0.8025
Hinrichs C et al. [42]	MRI	SVM + Linear Program boost (LP) + voxel-level engineered features	183 (AD + NC)	0.82
Suk H I et al. [21]	MRI	Deep Boltzmann Machine	93 AD/101 NC	0.9238
	PET			0.9220
Our proposed methods	MRI	FCN + SA	307 AD/243 NC	0.9788
		FCN + EA + softmax		0.9851
		FCN + EA + double normalization		0.9873

## Data Availability

Not applicable.
